# Controlling oleogel crystallization using ultrasonic standing waves

**DOI:** 10.1038/s41598-020-71177-6

**Published:** 2020-09-02

**Authors:** Fabio Valoppi, Ari Salmi, Miika Ratilainen, Luisa Barba, Tuomas Puranen, Oskari Tommiska, Petteri Helander, Jesse Heikkilä, Edward Haeggström

**Affiliations:** 1 Department of Food and Nutrition, University of Helsinki, Agnes Sjöbergin katu 2, P.O. Box 66, 00014 Helsinki, Finland; 2 Faculty of Agriculture and Forestry, Helsinki Institute of Sustainability Science, University of Helsinki, 00014 Helsinki, Finland; 3 Electronics Research Laboratory, Department of Physics, University of Helsinki, Gustaf Hällströmin katu 2, P.O. Box 64, 00014 Helsinki, Finland; 4 Istituto di Cristallografia, Consiglio Nazionale Delle Ricerche, 34100 Trieste, Italy

**Keywords:** Engineering, Physics, Applied physics, Materials science, Soft materials

## Abstract

Oleogels are lipid-based soft materials composed of large fractions of oil (> 85%) developed as saturated and hydrogenated fat substitutes to reduce cardiovascular diseases caused by obesity. Promising oleogels are unstable during storage, and to improve their stability careful control of the crystalline network is necessary. However, this is unattainable with state-of-the-art technologies. We employ ultrasonic standing wave (USSW) fields to modify oleogel structure. During crystallization, the growing crystals move towards the US-SW nodal planes. Homogeneous, dense bands of microcrystals form independently of oleogelator type, concentration, and cooling rate. The thickness of these bands is proportional to the USSW wavelength. These new structures act as physical barriers in reducing the migration kinetics of a liposoluble colorant compared to statically crystallized oleogels. These results may extend beyond oleogels to potentially be used wherever careful control of the crystallization process and final structure of a system is needed, such as in the cosmetics, pharmaceutical, chemical, and food industries.

## Introduction

Oleogels are lipid-based materials that contain 85%–99.5% liquid oil trapped in a network of structuring molecules called oleogelators^1^. Oleogels were developed during the last 15 years as saturated and hydrogenated fat substitutes^2^. Saturated fats are used in the food, cosmetics, and pharmaceutical industries due to their ability to form solid and crystalline structures at room temperature. These crystalline structures are employed as delivery and protective systems and structuring agents^3^. However, excessive consumption of saturated fats correlates with obesity that in turn causes cardiovascular diseases, metabolic syndrome and type-2 diabetes^4–6^. Obesity is a global problem. In 2014, 2.5 billion adults and 41 million children worldwide were overweight or obese; these numbers have doubled since 1980^7^. The annual healthcare costs related to treating diseases caused by/related to obesity is 60 billion euros in Europe^8^ and 210 billion dollars in the USA^9^.

Lowering the intake of saturated fats, for example, by using oleogels rich in polyunsaturated fatty acids can help reduce cardiovascular diseases caused by obesity. Oleogels can be prepared using direct^1^ and indirect^10^ methods. Indirect methods are foam, emulsion and solvent exchange and aerogel templating where proteins or polysaccharides are used to prepare the scaffold in which oil is absorbed/retained^10^. The direct method makes use of self-assembling molecules (e.g. monoglycerides, waxes, fatty acids, fatty alcohols, ethyl cellulose, phytosterols, phytosterol esters, etc.) to gel the oil^1^. Structuring agents are dispersed into the oil, and then a heating and a cooling step are successively applied. The oleogelators rearrange themselves during the cooling step to form a crystalline/polymeric network. The network entraps the oil and gels the system^11^. The direct method is most common because it is simple, needs no specific equipment, requires little energy during oleogel preparation and is industrially scalable^12^.

A disadvantage is that oleogels can release oil during storage due to molecular rearrangements. Unfortunately, this prevents these materials from becoming the ‘fat of the future’^2,11,13–15^. Much effort has been directed to improving oleogel performance by modifying the formulation and processing^16–18^. The cooling rate and shear force have been used to try to improve the ability of oleogels to retain oil^15,19–23^. In systems containing saturated fat, the application of shear force during crystallization aligns the crystals and decreases the oil migration rate due to a more densely packed crystal network (increased tortuosity of the system). This makes these systems more stable^24,25^. However, applying shear force to oleogels often reduces their ability to retain oil because small crystals with fewer junction zones among them are formed^20,21^. Recently, high-intensity ultrasound (HIU; high-power acoustic waves with a frequency above 20 kHz) have been used to tailor the mechanical and functional properties of saturated fats and oleogels^22,23,26^. Unfortunately, HIU creates streaming and particle fractionation effects due to transient cavitation, and this leads to uncontrolled modification of the oleogelator crystalline network. To improve the storage stability of oleogels, fine control of the assembling of the crystalline network is necessary. Such control can be achieved by exposing the oleogels to ultrasonic standing waves (US-SWs).

USSWs oscillate in place and are produced when a wave is confined within boundaries. Ultrasound travelling into a medium and reflecting off a surface interferes with the incident wave, creating regions of no displacement (nodes) and regions of maximum displacement (anti-nodes). This forms a USSW field. The acoustophoretic forces exerted by the USSWs can orient fibbers in aqueous media^27^, levitate objects^28^ and orient molecules^29^, microparticles^30^, nanowires^31^, and cells^32^ across many length scales (e.g. nano, micro, and macro) without damaging the samples.

We use USSWs to direct the crystallization of monoglyceride-, sunflower wax-, and candelilla wax-containing oleogels and achieve fine control of their microstructure. We chose monoglyceride and waxes because they are promising oleogelators in terms of possible applications^16,33^. We organized the work into four different parts: (i) design, simulation and development of technology made in-house, (ii) the effect of frequency and cooling rate on the crystallization of oleogels with the development of a theoretical model, (iii) the effect of oleogelator concentration and type and (iv) the effect of USSW treatment on liposoluble colorant migration kinetics. USSWs can potentially be used as a platform technology to obtain fine control of the crystallization process of systems that contain a growing crystalline network.

## Results and discussion

### Design, simulation, and test of the experimental chamber

**Figure 1:**
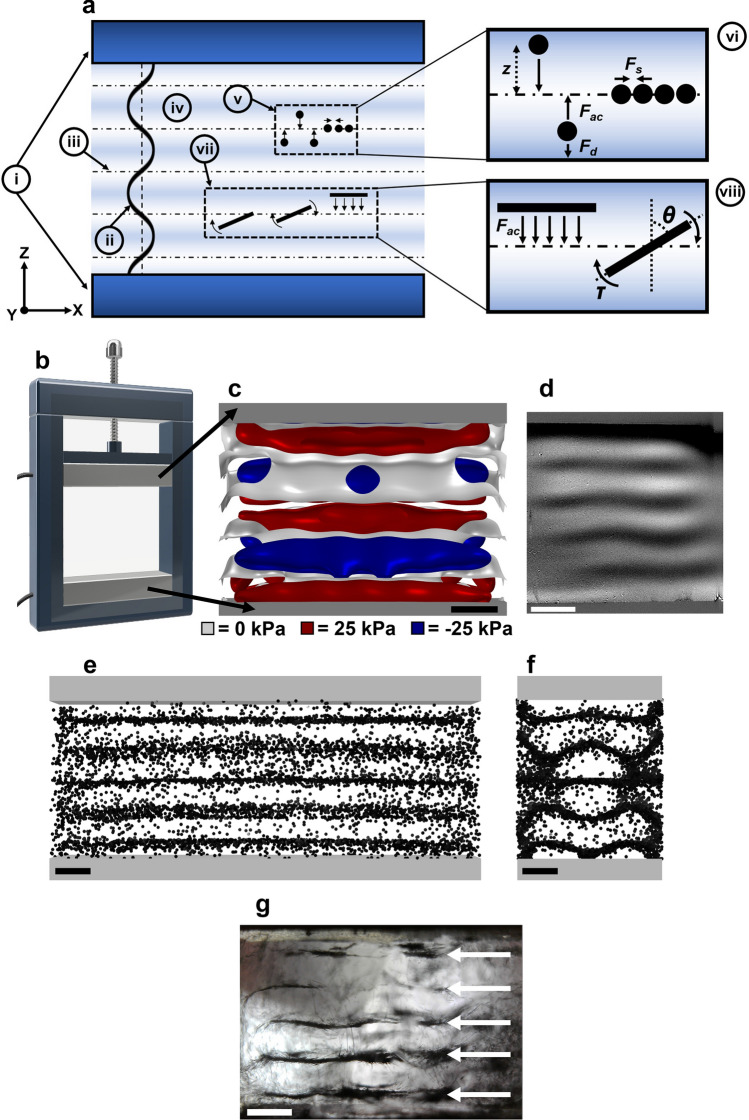
(**a**) Physical principles governing the behavior of particles in USSW fields: (i) piezoceramic elements, (ii) sinusoidal wave generated by the lower piezoceramic element, (iii) nodal plane corresponding to a pressure minimum, (iv) anti-nodal plane corresponding to a pressure maximum, (v) and (vi) behavior of spherical particles in the USSW field, with arrows indicating forces acting on the particles (*F*_*ac*_ = acoustic radiation force, *F*_*d*_ = drag force, *F*_*s*_ = secondary radiation force, *z* = distance between the center of the particle and the nodal plane), (vii) and (viii) behavior of rod/platelet-like particles in the USSW field, with arrows indicating the forces acting on the particles (*θ* = angle between the normal of the nodal plane and the major axis of the rod, *τ* = torque). (**b**) Design of the experimental chamber. (**c**) Finite element simulation of the ultrasonic standing field in the 1 MHz experimental chamber (top and bottom grey parts are the piezoceramic elements). (**d**) Schlieren imaging of the USSW field in the 1 MHz experimental chamber, with oil used as the medium. (**e**) Front view (XZ plane) of particle movement simulation for 10,000 spherical particles of 100 µm diameter made of monostearin after 10 s in the 1 MHz USSW field. (**f**) Corresponding lateral view (YZ plane) of (**e**). (**g**) Image of 30 µm–175 µm long carbon rods dispersed in rapeseed oil and subjected to a 1 MHz USSW field; arrows indicate the formed layers of carbon rods. In (**c**–**f**), the scale bar represents 1 mm along the vertical direction.

Figure 1a shows the physical principles governing the behavior of particles in USSW fields. These fields feature nodal and anti-nodal planes. Between these planes, there is a radiation pressure gradient that exerts a force on any particle whose acoustic impedance differs from that of the immersion medium. The primary force that the planar standing field generates is the acoustic radiation force (F_ac_)^27,32,34^:

1 $${F}_{ac}=-{V}_{p}k{E}_{ac}\varphi \mathrm{sin}\left(2kz\right),$$

where *V*_*p*_ is the volume of the particle, *k* is the wave number (2*π*/*λ* where *λ* is the ultrasound wave length), *E*_*ac*_ is the maximum acoustic energy density, *z* is the distance between the particle and the nodal or anti-nodal plane, and *φ* is the acoustic contrast factor, defined as:

2 $$\varphi =\frac{5\widehat{\rho }-2}{2\widehat{\rho }+1}-\widehat{\beta },$$

where $\widehat{\rho }$ is the ratio of the density of the particles to that of the medium, and $\widehat{\beta }$ is the ratio of the compressibility of the particles to that of the medium.

*F*_*ac*_ pushes the particle towards the nodes or anti-nodes, and the force experienced by the particle changes its magnitude and direction based on the position of the particle with respect to the nodal or anti-nodal planes^27^. Particles move towards the nodal planes if *φ* is positive. In contrast, particles move towards the anti-nodes if *φ* is negative. During their movement towards the nodal plane, a drag force opposes the particle movement due to, for example, the viscosity of the medium^27,34^. When the particles arrive at the nodal plane, a secondary force acts on the particles and pushes them together. This force is known as secondary radiation force (*F*_*s*_)^34^. In the case of non-spherical particles, the particles may feel a torque (*τ*). This torque is generated by the inhomogeneous radiation pressure acting along the long axis of the particles^27,32^. For instance, a rod-shaped particle can partially lie in the nodal plane while the remaining part is in the anti-nodal plane, and therefore the particle rotates. The force acting on the particle is proportional to *cos*(2*θ*) of the deflection angle *θ*. Only at *θ* = 90° (rod horizontally oriented in Fig. 1a) is the orientation stable, and no rotation takes place. If the particle size is larger than λ/2, the formation of macrostructures is weakened because their characteristic length scale is λ/2^27^, and the acoustic forces cease to rapidly increase with particle size, unlike viscous forces. At the same time, the particle must be large enough (e.g. in the micrometer range); otherwise, the drag forces (F_d_, Fig. 1a) prevent translation in any sensible time. Because *F*_*ac*_ and *F*_*d*_ are proportional to particle volume (*V*_p_) and area (*A*_p_), respectively, particles with small a surface-to-volume ratio (*A*_p_/*V*_p_ < 1 µm^−1^) are more easily moved inside a USSW field.

Based on these considerations, we designed an experimental chamber featuring two ultrasonic transducers able to generate a prescribed standing field (Fig. 1b). The upper transducer is movable so that the number of nodal planes can be selected according to Eq. 7 (“Materials and methods”). We then simulated the standing field in the experimental chamber. As an example, we report in Fig. 1c on the simulation of a 1 MHz ultrasonic standing field generated by emitting ultrasonic waves from the lower transducer placed 3.8 mm apart from the top transducer (corresponding to 2.5 λ) in oil. The standing field is composed of nodal planes (white) between areas of alternating positive (red) and negative (blue) pressure. Similar results were obtained in the simulation of a 2 MHz chamber (Fig. S1a, supporting information).

We then visualized the standing field in the real chamber using a Schlieren setup, which confirmed the presence of the pressure field in the chamber in holding oil (Figs. 1d and S1b, supporting information). In Schlieren experiments, the dark and bright areas are proportional to the derivative of the refractive index in the direction of the applied spatial filter^35^; that is, a different contrast between areas is related to differences in pressure, and the nodal and anti-nodal planes lie between the dark and bright areas where the intensity is equal to that of the background. To gain insight into the behavior of particles in the standing field, we ran particle tracing simulations in the 1 MHz and 2 MHz standing field chambers using 10,000 spherical particles of 100 µm diameter made of carbon or monostearin (Videos 1 and 2). In both cases, the particles moved towards the nodal planes as expected, with the acoustic contrast factor being positive in both cases (simulation for carbon particles not shown). Boundary effects are visible on the lateral sides of the simulated experimental chamber at both 1 MHz and 2 MHz, where bands of particles are less defined compared to the central part (Figs. 1e and S1c, supporting information, respectively). Bands are continuous along the y direction at both frequencies (Figs. 1f and S1d, supporting information), even if some irregularities in shape are found at 1 MHz. We finally studied the behavior of carbon rods with a length of 30 µm–175 µm immersed in rapeseed oil and subjected to 1 MHz and 2 MHz standing fields. The USSW field pushed the carbon rods to the nodal planes (cf. Fig. 1e,g), forming horizontal bands of stacked carbon rods (Fig. 1g). The carbon rods experienced a torque that aligned them horizontally (in agreement with the data of Yamahira, Hatanaka, Kuwabara and Asai^27^) on polystyrene rods dispersed in an aqueous solution.

### Effect of frequency and cooling rate on oleogel crystallization

We studied the effect of 0 MHz (control samples), 1 MHz, 2 MHz, and 4 MHz USSW fields on the crystal network formation of 5% monoglyceride-containing oleogel crystallized at cooling rates of 1 °C min^−1^ and 10 °C min^−1^.

**Figure 2:**
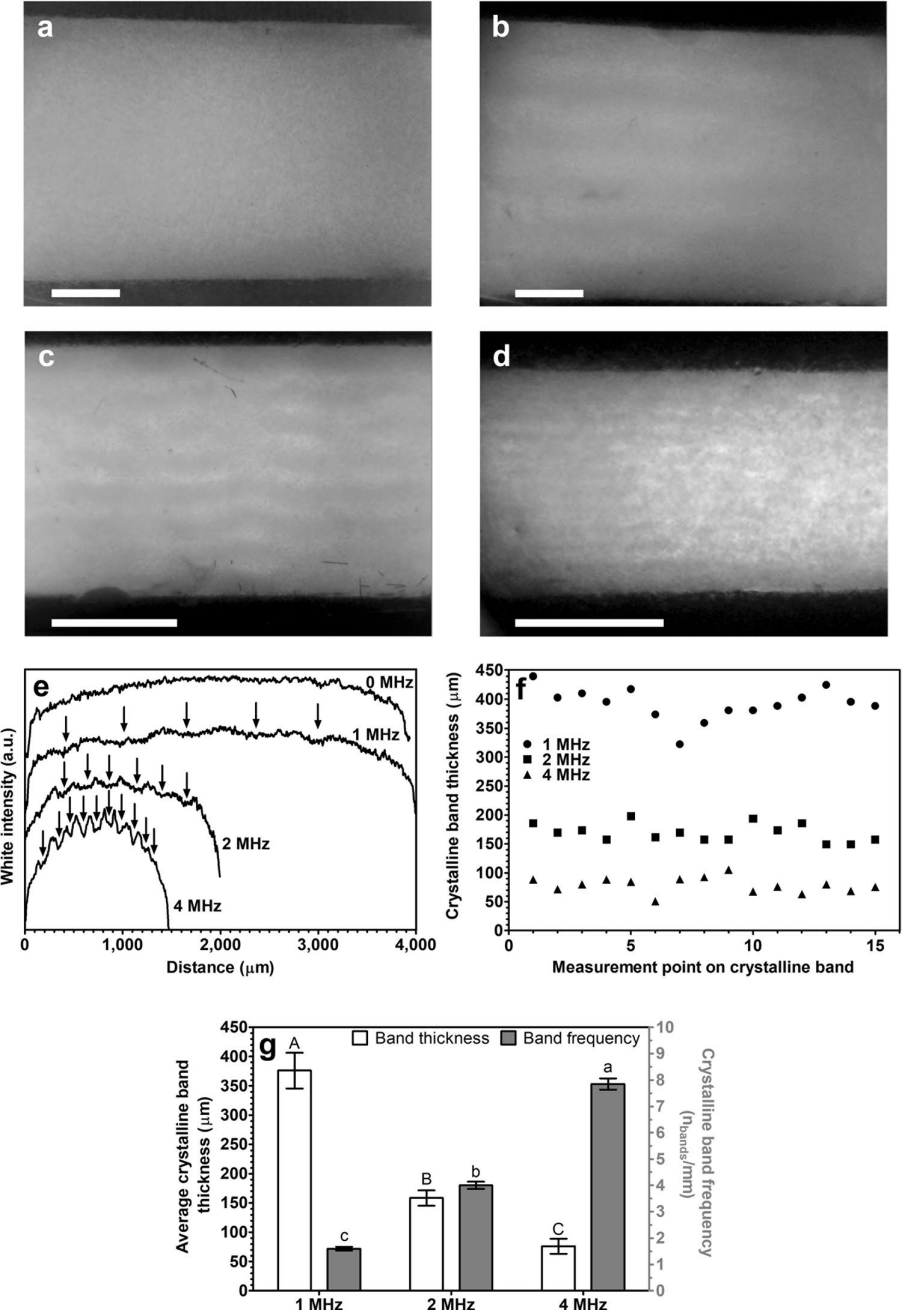
(**a**) Photograph: oleogel statically crystallized, control sample (0 MHz). (**b**) Photograph: oleogel crystallized in a 1 MHz US-SW field. (**c**) Photograph: oleogel crystallized in a 2 MHz US-SW field. (**d**) Photograph: oleogel crystallized in a 4 MHz US-SW field. (**e**) White intensity recorded along the Z direction (vertically) from a photograph as a function of the distance from the top transducer for the control sample (0 MHz) and sonicated samples (1 MHz, 2 MHz, and 4 MHz). Curves were shifted vertically for clarity. Arrows indicate the presence of a crystalline band. (**f**) Thickness of a crystalline band measured on 15 points along the X direction (horizontally) from a photograph of sonicated samples (1 MHz, 2 MHz, and 4 MHz). (**g**) Average crystalline band thickness and normalized number of crystalline bands per mm for sonicated samples (1 MHz, 2 MHz, and 4 MHz) analyzed from photographs. Data is expressed as mean ± standard deviation based on n = 2 experimental replicates × 8–12 repeated measurements for the band thickness and n = 2 experimental replicates for the crystalline band frequency. Values with different uppercase or lowercase letters are statistically different (p < 0.05). In (**a**), (**b**), (**c**), and (**d**), oleogels that contain 5% monoglyceride in rapeseed oil are crystallized at 1 °C min^−1^. The top and bottom dark areas are the piezoceramic transducers, and the scale bars are 1 mm.

The application of USSW fields on oleogels modified the microscopic structure during crystalline network formation. Treated samples appeared inhomogeneous (Fig. 2b–d) compared to the homogenous structure of the control sample (Fig. 2a). Sonicated samples feature alternated/stacked dark and light horizontal bands. The density of the crystalline material in the bands is proportional to the darkness of the bands. Darker layers contain more crystalline material than lighter layers. The crystalline bands developed through the entire thickness of the sample (Z direction, Fig. 2e). The thickness of the bands was uniform (Fig. 2f) and inversely proportional to the frequency applied (Fig. 2g). Moreover, the number of crystalline bands was directly proportional to the applied frequency (Fig. 2g). During crystallization, monoglyceride crystals were pushed towards the nodal planes (cf. Figs. 1e and 2b and Video 3) by the pressure gradient forming alternating dense and sparse bands. Applying a 10 °C min^−1^ cooling rate instead of a 1 °C min^−1^ did not statistically alter the band thickness or the number of bands with the same USSW frequency (p > 0.05, cf. Figs. 2 and S2, supporting information). Microstructural changes are known to affect the mechanical properties and stability of oleogels^15,19–21^; therefore, the bands formed in oleogels might give different mechanical properties and might affect the stability of the oleogel.

Elongated objects such as cells^32^ and polystyrene fibers^27^ in aqueous solutions subjected to a USSW field accumulate in the nodal planes and form bands of densely packed material, similar to the formation of monoglyceride crystals in oil. The emergence of bands is due to the saturation of the nodal plane by the crystalline material. After saturation, crystals are continuously pushed and occupy the closest position to the nodal plane, thus forming a band. Moreover, oleogelator crystals change their dimensions (and volume) during crystallization, and the acoustic force acting on them increases during crystallization (Eq. 1). Indeed, it is known that oleogelator crystals show a hierarchical structure similar to that of saturated fats^36,37^. Hence, there is continuous crystal growth from nanocrystals to microcrystals, and eventually the crystals form a macroscopic network. Therefore, during crystallization, the forces acting on monoglyceride crystals increase and speed up the translation of the crystals to the nodal plane. Additionally, a fast cooling rate produces a high number of nucleation points, leading to a high number of small microcrystals with respect to the case where one applies a slow cooling rate^15,19,38^. In our case, possible differences in microcrystal dimensions^38^ did not modify the final outcome, proving that USSWs can be applied as a robust method when employing commonly used cooling rates for oleogel preparation^15,17,19,38,39^.

To understand the dynamic behavior of crystalline platelets in oil subjected to the USSW field, we carried out a mathematical derivation assuming crystals to be disk-shaped particles (ratio between disk diameter and thickness equal to or greater than 10. We assumed the crystals to be disk-shaped because this geometrical shape can approximate the flat crystalline platelets observed in oleogels containing monoglycerides^21,40^, and disks are simpler to model compared to platelets. We present the equations used to approximate the median time necessary for the crystalline platelets to translate and rotate in the standing field as a function of the acoustic pressure experienced. The derivation of the equations is presented in the supporting information.

Equations 3 and 4 of motion for a single crystal residing in an ultrasonic standing wave are:

3 $$m\frac{{\partial }^{2}z}{\partial {t}^{2}}=B\frac{\partial z}{\partial t}+{K}_{a}{\mathrm{sin}}2kz$$

4 $$I\frac{{\partial }^{2}\theta }{\partial {t}^{2}}=\beta \frac{\partial \theta }{\partial t}+{\kappa }_{a}\mathrm{sin}2\theta$$

Equation 3 features the position $z$ and stiffness ${K}_{a}$, whereas Eq. 4 features the orientation $\theta$ and rotational stiffness ${\kappa }_{a}$. Due to the large dynamic viscosity of the fluid, the mass $m$ and the moment of inertia $I$ are negligible, and the behavior is dominated by the drag coefficients $B$ and $\beta$. The effect of gravity is neglected for the same reason. When calculating the numerical values of the coefficients, we assumed the crystal to be disk-shaped with an aspect ratio equal to or greater than 10. By solving the differential equations, we find that the crystals orient themselves perpendicularly to the standing wave (horizontally as depicted in Fig. 1a) and slowly travel towards the pressure node at $z=0$. We derived the median transport (Eq. 5) and rotation (Eq. 6) time. The model does not consider the crystal growth rate. However, in the case of platelets of 50 µm–100 µm in size, the translation time is comparable to that observed during the experiments:

5 $${t}_{\mathrm{tra}}\left({V}_{p},p\right)\approx 1.2\frac{B}{{K}_{a}\cdot k}$$

6 $${t}_{\mathrm{rot}}\left({V}_{p},p\right)\approx 1.2\frac{\beta }{{\kappa }_{a}}$$

**Figure 3:**
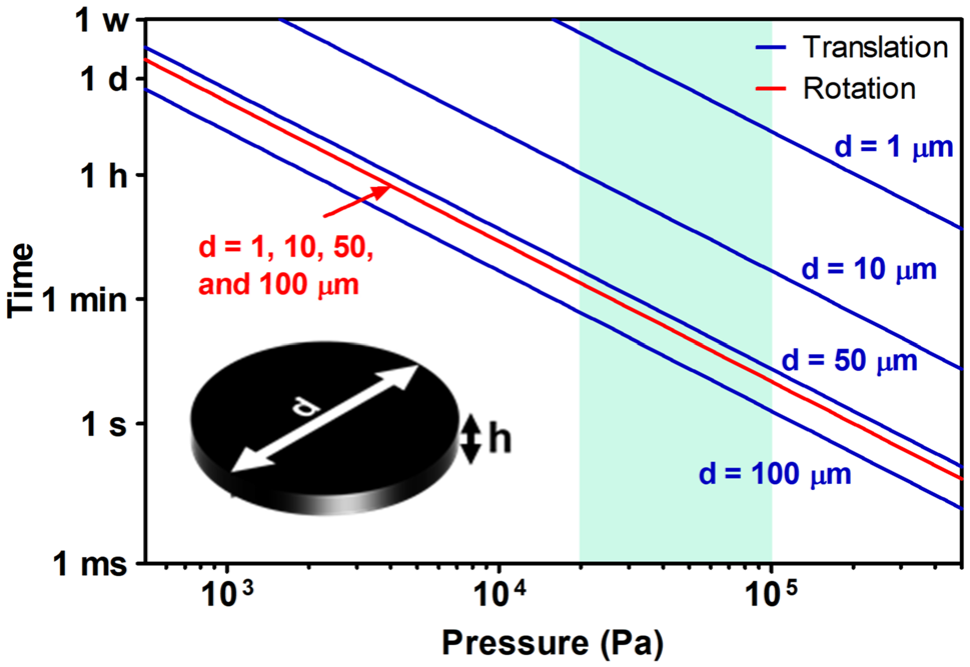
Theoretical estimated characteristic time for rotation (orientation, t_rot_) and translation (t_tra_) of disk-shaped monostearin crystals with diameters of 1 µm, 10 µm, 50 µm, and 100 µm and an aspect ratio (d/h) equal to or greater than 10 in a 1 MHz (936 kHz) US-SW field. The highlighted area corresponds to the estimated pressure amplitude based on the time needed during experiments to form bands, taking into consideration platelets of 50 µm–100 µm in size.

In Fig. 3, the median travel time is shown as a function of the acoustic pressure generated in a 1 MHz USSW field at the anti-node. The curves show that the rotation time is independent of the crystal size, whereas the translation time is shorter for large crystals. Increasing the pressure $p$ reduces the characteristic times considerably, $t\propto {p}^{-2}$. Crystalline platelets grow during cooling, which reduces their translation time as depicted by the four different diameters considered in Fig. 3. The predicted time is on order of minutes to seconds for the pressure values estimated in the current experiments (20 kPa–100 kPa), which agreed with the observed behavior. Increasing the frequency of the USSW field reduces the transportation time (cf. Figs. 3, S3a and S3b, supporting information). This is because the nodal planes are closer at higher frequencies thus reducing *z*, and *K*_*a*_ increases due to higher acoustic force. The rotation time remains unaffected.

**Figure 4:**
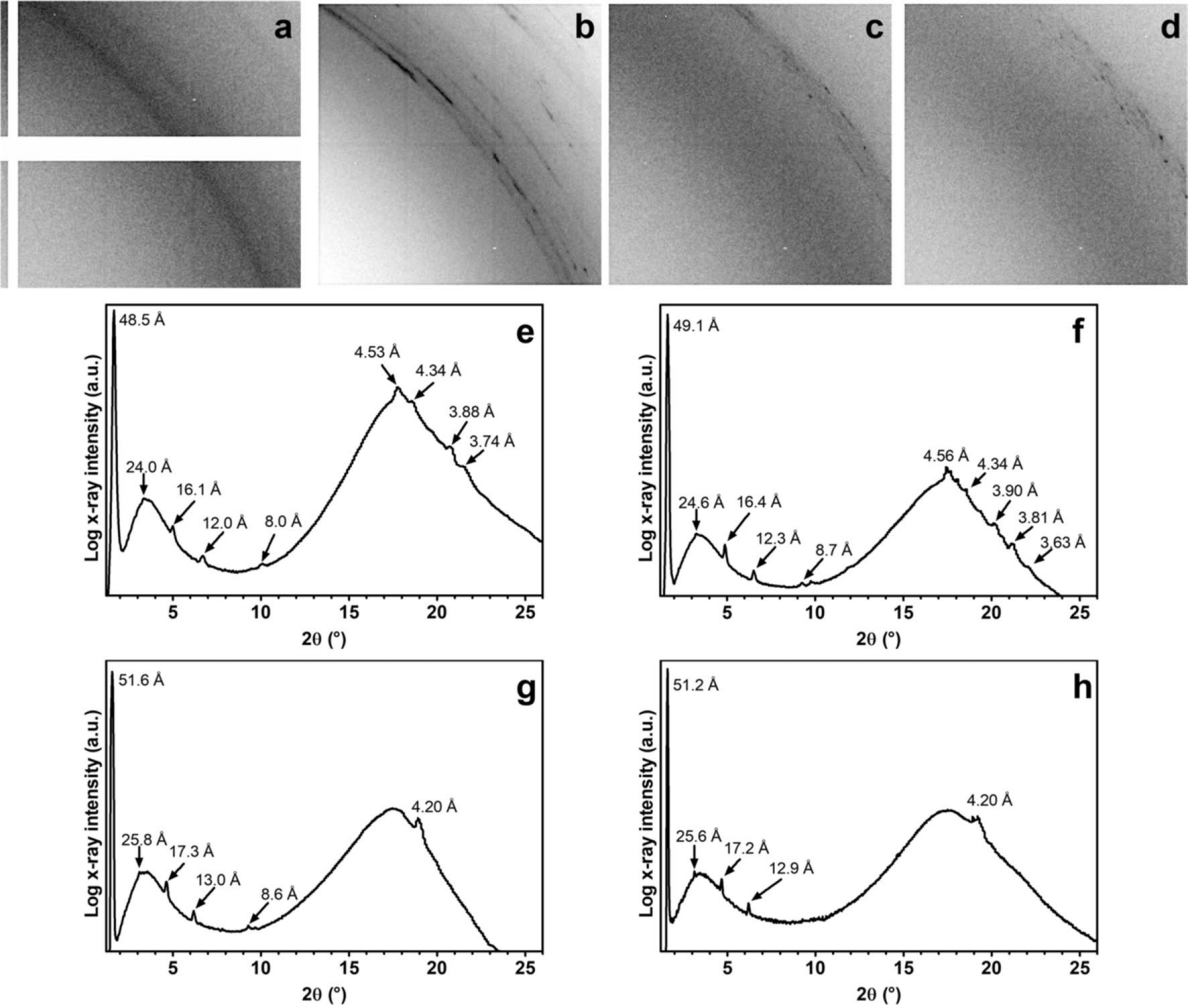
Selected area of bidimensional diffraction patterns in the wide-angle X-ray diffraction region between 12.3° and 21.7° 2θ (diagonal), showing portions of the Debye–Scherrer diffraction rings for (**a**) statically crystallized oleogels (0 MHz) and oleogels crystallized at (**b**) 1 MHz, (**c**) 2 MHz, and (**d**) 4 MHz. Integrated patterns with relevant peak position of (**e**) statically crystallized oleogels (0 MHz), and oleogels crystallized at (**f**) 1 MHz, (**g**) 2 MHz, and (**h**) 4 MHz. All figures refer to oleogels containing 5% monoglyceride in rapeseed oil crystallized at 1 °C min^−1^.

Finally, we studied the nano- and microstructural organization of monoglyceride crystals at the nodal and anti-nodal planes. We scanned selected samples vertically and horizontally using synchrotron light X-ray diffraction. Figure 4a–d show a selected area of the diffraction pattern in the wide-angle X-ray diffraction region between 12.3° and 21.7° 2θ (diagonal). The figures show portions of the Debye–Scherrer diffraction rings for the statically crystallized oleogel (0 MHz, Fig. 4a) and oleogels crystallized at 1 MHz, 2 MHz, and 4 MHz (Fig. 4b–d, respectively). Whole diffraction patterns are shown in Fig. S4 (supporting information). In general, all treated samples showed the formation of microcrystals revealed by the presence of the dark spots on the diffraction rings (Fig. 4b–d). In contrast, the statically crystallized sample showed homogeneous diffraction rings indicating that no microcrystals were formed. We determined that microcrystals were more abundant in the dense crystalline bands than in the sparse bands (data not shown). The nanostructural organization of monoglycerides was triclinic β-form with a lamellar thickness of 48 Å–49 Å for oleogels statically crystallized (Fig. 4e), in agreement with previously reported data^21,41,42^. Similar results were obtained for the oleogels crystallized at 1 MHz (Fig. 4f), whereas by increasing the USSW field frequency to 2 MHz and 4 MHz, monoglycerides tended to crystallize in the less thermodynamically stable hexagonal α-form with a lamellar thickness of 51.5 Å (Fig. 4g,h). USSW fields with higher frequencies (2 MHz and 4 MHz) can disturb monoglyceride crystallization at the nanostructural level, forming less stable polymorphic forms. Therefore, USSW fields can affect both micro- and nanostructural levels depending on the selected frequency.

Using synchrotron light X-ray diffraction, we confirmed that the dense bands observed in Figs. 2 and S2 were richer in monoglyceride crystals compared to the sparse bands. This was revealed by the trend in intensity of the monoglyceride lamellar structure 001 reflection (d_001_) recorded on the dense bands during vertical scans (Fig. S5a, supporting information). Oleogels crystallized under the USSW field showed peaks and troughs in the diffracted X-ray beam intensity compared to the statically crystallized oleogel. Moreover, we determined that the dense crystalline bands have a constant abundance of crystalline monoglycerides in the X direction (horizontal) (Fig. S5b, supporting information).

### Effect of oleogelator concentration and type

We tested selected frequencies (1 MHz and 2 MHz) on oleogels containing 2.5%, 10%, and 20% monoglycerides or 2.5% and 5% candelilla or sunflower wax.

**Figure 5:**
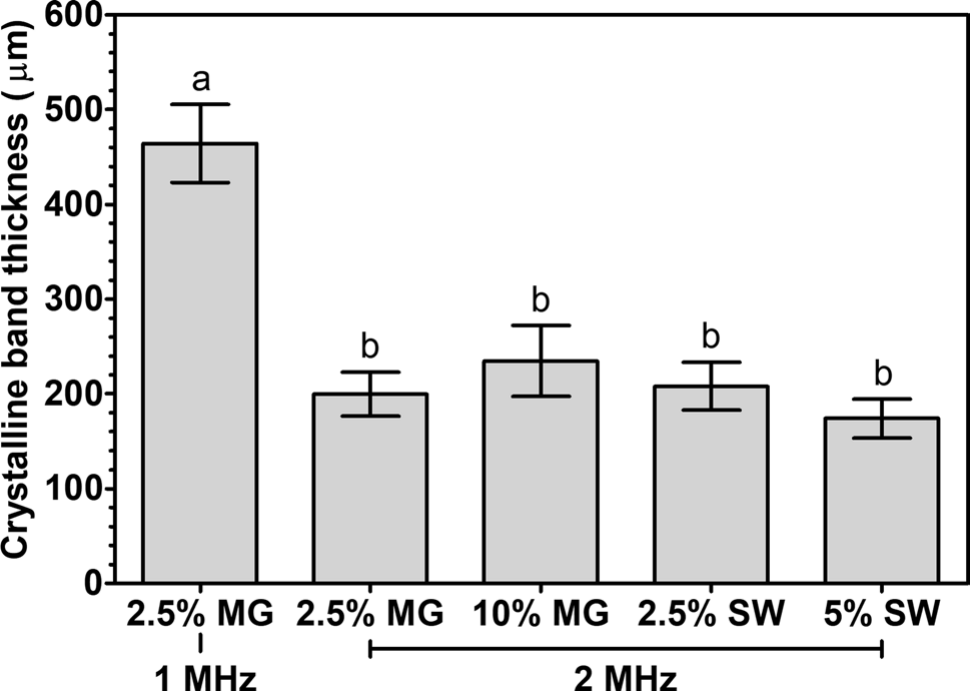
Average crystalline band thickness analyzed from photographs for oleogels containing 2.5% and 10% monoglycerides and 2.5% and 5% sunflower wax in rapeseed oil, sonicated at 1 MHz and 2 MHz during cooling at 10 °C min^−1^. Data is expressed as mean ± standard deviation based on n = 2 experimental replicates × 5 repeated measurements. Values with different letters are statistically different (p < 0.05).

We obtained alternating horizontal bands with high and low concentrations of crystalline material in all samples (visually evaluated). The results of the thickness of the crystalline bands are shown in Fig. 5. The thickness of the crystalline bands for samples sonicated at 2 MHz did not statistically differ (p > 0.05) from each other or from those measured in 5% monoglyceride oleogels treated at 2 MHz (cf. Figure 2g). However, the thickness of the crystalline bands obtained in 2.5% monoglyceride-containing oleogel treated at 1 MHz was greater than 5% monoglyceride-containing oleogel sonicated at the same frequency (p < 0.05). We were unable to calculate the thickness of crystalline bands in some samples after crystallization (i.e. 10% monoglyceride treated at 1 MHz, 20% monoglyceride treated at 1 MHz and 2 MHz and all samples containing candelilla wax treated at 1 MHz and 2 MHz). This is because of some limitations of our current setup, the tightly packed crystal network formed in candelilla wax oleogels compared to sunflower wax oleogels^43^ and the dense network of oleogels containing high concentrations of monoglycerides^38,41^. However, during crystallization we also observed the formation of bands in the latter samples (Fig. S2, supporting information).

Different concentrations and types of oleogelators modify the crystalline network density and oleogelator platelet dimensions^15,38,43^. For instance, candelilla wax forms a densely packed crystalline network composed of small oleogelator microcrystals compared to the less densely packed network formed by the bigger sunflower wax microcrystals^43^. However, irrespective of these differences in oleogel structure we demonstrated that the USSW field can modify the crystalline microstructure of the oleogelator network. This indicates that this technology can be used as a platform technology to modify the microstructure of the crystalline network in different oleogels.

### Colorant migration

Finally, we tested the effect of the USSW-modified oleogels’ microstructure on colorant migration.

**Figure 6:**
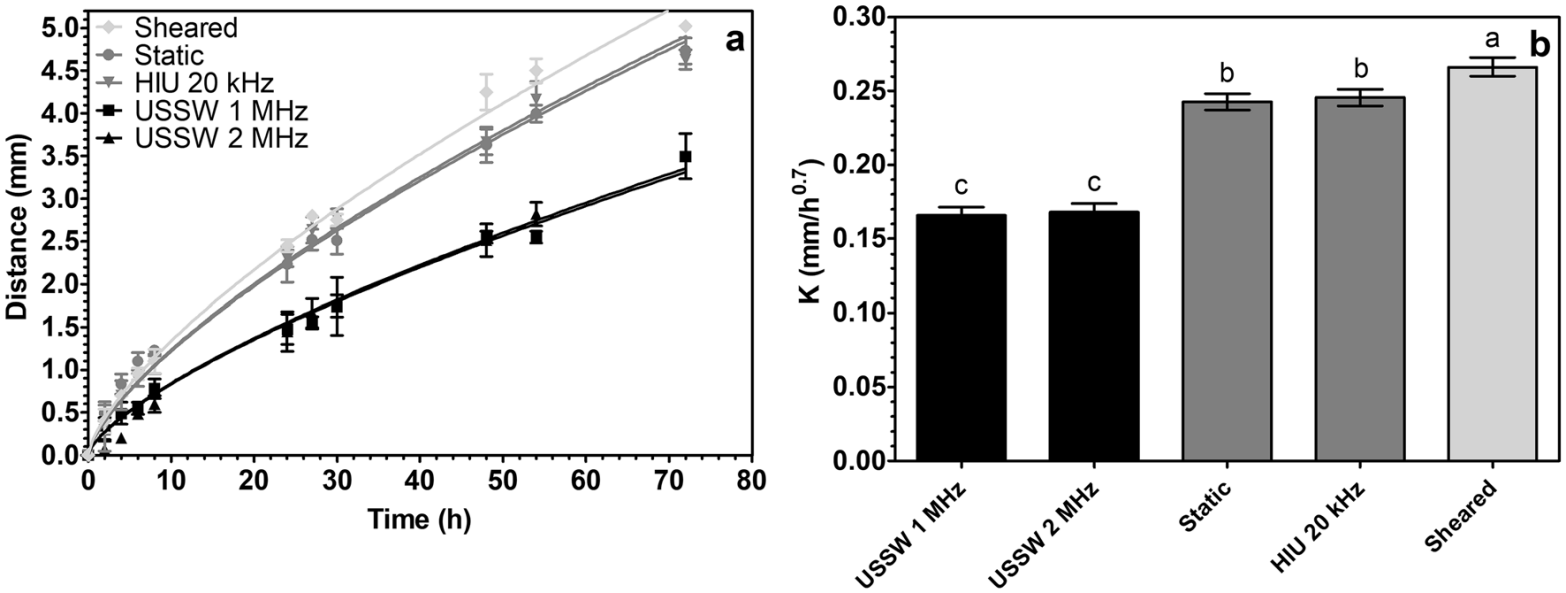
(**a**) Migration of colorant expressed in mm inside 5% monoglyceride oleogel crystallized at 10 °C/min as a function of time for samples statically crystallized, crystallized under HIU, sheared, and crystallized under a USSW field. Data is expressed as mean ± standard deviation based on n = 3 experimental replicates × 3 repeated measurements. (**b**) Migration rate constants ± 95% confidence interval (error bars) expressed in mm/h^0.7^ for the samples shown in (**a**). Values with different letters are statistically different (p < 0.05). The temperature was 22 ± 2 °C.

Figure 6a shows the migration of colorant into 5% monoglyceride oleogel crystallized at 10 °C min^−1^ along the Z direction (vertically) as a function of time for samples statically crystallized, crystallized under HIU, sheared, and crystallized under a USSW field. We selected 5% monoglyceride oleogel due to the clear bands observed during USSW treatment. The colorant diffuses slower in samples crystallized under a USSW field compared to the state-of-the-art technology used to tailor oleogel and fat structures. In the figure, data were fitted using an exponential equation (Eq. 8) and a data analysis paragraph. After a preliminary fitting, the reaction order exponent *n* was fixed to 0.7, as it was the average value calculated among samples. This allowed us to compare the migration rate constant for the different samples. Figure 6b shows the migration rate constants with their 95% confidence interval (error bars) expressed in mm/h^0.7^ for the corresponding samples shown in Fig. 6a. Figure 6b shows that an USSW retards the migration of the colorant inside oleogel samples because *K* is smaller for USSW- treated samples compared to the other samples (HIU, static, sheared). This could be due to the formation of layers of crystalline oleogelators, as shown in Fig. 2. The obtained layers act as physical barriers that slow down the migration of the colorant. No statistical differences (p > 0.05) were found in the colorant migration rate constant between 1 MHz- and 2 MHz-treated oleogels. This could be due to the actual thickness of the dense crystalline layer that the colorant has to go through. Indeed, the sum of the thickness of the dense crystalline bands in, for example, 1 mm in the vertical direction (Z direction) for both frequencies is 560 ± 30 µm in 1 MHz and 680 ± 86 µm in 2 MHz. Because the crystalline bands act as physical barriers, the thickness of the barrier that the colorant has to go through is similar in both samples, leading to a similar rate constant. Colorant migration in oleogels has been correlated to oil migration^44^ and oxygen migration^45^. Further studies are needed to verify the barrier properties of oxygen in the crystalline bands formed during USSW treatment.

## Conclusion

We ultrasonically pushed the crystallization domain into a band-like topology. The thickness of the crystalline bands depended on ultrasound frequency, as expected from standing wave theory. The behavior of crystallizing oleogels under the USSW field was predicted using the finite element method of simulation, and the dynamics of oleogelator crystals in the USSW field was derived by modelling the acoustophoretic force and drag force acting on disk-shaped particles. We demonstrated that in our experimental conditions a USSW field can be applied as a robust method for oleogel preparation independent of oleogelator type (monoglyceride, sunflower wax, candelilla wax), oleogelator concentration (between 2.5 and 20%) and cooling rate (1 °C min^−1^ and 10 °C min^−1^). Moreover, the reduced migration of a liposoluble colorant in the ultrasonically obtained microstructure suggests that USSWs can induce the formation of physical barriers that might be further used to improve oleogel performance.

These results suggest that USSW fields are a promising technology to modify the crystalline network of oleogels. USSW field technology is not limited to oleogels but can potentially be used as a platform technology in the cosmetics, pharmaceutical, chemical, and food industries where careful control of the crystallization process and the final structure of the system is needed.

## Materials and methods

### Materials

Rapeseed oil was purchased at a local supermarket, Myverol 18–04 K saturated monoglyceride (fatty acid composition: 42–48%, C16:0, 50–60% C18:0; melting point 68.05 ± 0.5 °C) was donated by Kerry Ingredients and Flavours (Bristol, UK). Sunflower and candelilla waxes were donated by Bionord AB (Stenungsund, Sweden). Red Oil O was purchased from Sigma-Aldrich (St. Louis, MO, USA). Acrylonitrile butadiene styrene and polylactic acid 3D printer materials were purchased from Ultimaker B.V. (Utrecht, The Netherlands). Hard piezoelectric ceramic Pz26 transducers with electrodes on both surfaces were purchased from Meggit Ferroperm Piezoceramics (Kvistgaard, Denmark).

### Oleogels

Oleogels were prepared by mixing rapeseed oil with increasing concentrations of monoglyceride (2.5%, 5%, 10%, and 20%) or wax (2.5% and 5%) at 80 °C or 90 °C in an oven for at least 15 min until the crystalline material was completely melted. During melting, the mixture was mixed several times. The molten system was then transferred into the preheated experimental chamber. All concentrations are expressed as mass percentage (% w/w).

### Experimental chamber

The experimental chambers had a variable treatment volume between 50 µL and 500 µL corresponding to 11 × 4.3 × 1 mm^3^–10 mm^3^ featuring two piezoelectric ultrasonic transducers (Fig. 1b). The bottom transducer was used as an active element to generate the ultrasonic waves, whereas the top one was used as a passive reflector so that a standing field was obtained. The top transducer was connected to an oscilloscope to monitor the acoustic pressure inside the chamber. The maximum amplitude was obtained when the distance (d) between the transducers satisfies (Eq. 7):

7 $$d=n\frac{\lambda }{2} (n=1, 2, 3, 4\dots ),$$

where λ is the wavelength of the ultrasound in oil, and *n* is the number of nodal planes in the experimental chamber. The chambers were 3D printed in acrylonitrile butadiene styrene using an Ultimaker 3. Ultrasonic transducers with nominal resonant frequency of 1 MHz, 2 MHz, and 4 MHz (thickness: 2 mm, 1 mm, and 0.5 mm, respectively) were cut to a rectangular shape of 10–10.5 × 3.5 mm^2^–4 mm^2^ using a low-speed saw mounted with a diamond blade. The two transparent windows in the experimental chamber used to monitor the process were obtained by sealing two glass cover slips (24 × 24 × 0.16 mm^3^–0.19 mm^3^, #1.5, Menzel Gläser, Braunschweig, Germany) with silicone (Elastosil E41 Transparent, Wacker Chemie, Munich, Germany) to the 3D printed body.

An Analog Discovery 2 (Digilent, Pullman, WA, USA) multi-function instrument was used for signal generation and to monitor the amplitude at the reflector. To drive the active transducer, a square wave was generated with a signal generator and amplified with a MOSFET pair (TC6320, Microchip, Espoo, Finland). The amplitude of the driving signal was controlled by an adjustable power supply (Elektro-Automatik GmbH & Co. KG, Viersen, Germany).

The temperature during USSW-assisted crystallization was monitored using a T-type thermocouple inserted directly into the sample. Samples were crystallized by cooling the molten oleogel to room temperature either in air at 10 °C min^−1^ or by using a custom-built temperature control device where the cooling rate was set to 1 °C min^−1^. The temperature control device comprises two Peltier elements (TEC1-12706, Hebei, Shanghai, China) that either cool or heat the sample depending on the desired cooling rate. The Peltier elements are driven by an H-bridge controlled by a microcontroller running a PID loop.

Carbon rods dispersed in rapeseed oil or molten oleogels were transferred into the experimental chamber. The chamber was preheated to 80 °C before adding oleogel, whereas experiments involving carbon rods were carried out at room temperature (20 °C). After the chamber was connected to the custom-built firmware, the top passive transducer mounted on the positioning arm was inserted into the chamber and sonication was immediately started. The height of the passive transducer and frequency of the active transducer were adjusted to obtain a standing field (maximizing the signal received by the passive transducer). In general, the height was 3.5 mm–4 mm, 2 mm–2.5 mm, and 1.5 mm for the chambers featuring 1 MHz, 2 MHz, and 4 MHz transducers, respectively. The amplitude of the driving signal was selected to maximize the organizing effect of the standing field (i.e. minimize the time needed to form the crystalline bands) without producing adverse effects, such as uncontrolled streaming based on preliminary trials. The voltage applied to the transducers was 25 V, 10 V, and 5 V for the chambers featuring 1 MHz, 2 MHz, and 4 MHz transducers, respectively. The excitation frequencies were in the range of nominal frequencies ± 0.1 MHz. During oleogel treatment, sonication was continued until the sample was crystallized (at least 5 min after the sample turned opaque). Oleogel control samples were prepared in the same way without applying US-SWs. The schematics of the setup are presented in Fig. S7 (supporting information).

### Finite element method computer simulations

The standing acoustic field and the particle movement were simulated using COMSOL Multiphysics version 5.4 finite-element method simulation software^46^. The standing acoustic field was simulated using COMSOL’s Pressure Acoustics, Frequency Domain interface. Piezoelectric effects and waves in solids were simulated using Electrostatics and Solid Mechanics interfaces. The particle movement was simulated using the Particle Tracing for Fluid Flow interface. All required couplings between different physics interfaces were done using COMSOL’s built-in multiphysics interfaces.

Two different 3D model geometries were studied, a chamber designed to be used with a 936 kHz (1 MHz) signal and a chamber designed to be used with a 2 MHz signal. Each chamber had two piezo elements, the active piezo that was driven with a voltage signal to create the standing field and the passive piezo acting as a reflector. The piezo elements were 10.5 × 3.5 × 2 mm^3^ and 10.5 × 3.5 × 1 mm^3^ for the 1 MHz and 2 MHz chambers, respectively. The distance between the active and the passive piezo was calculated to be 2.5 times the wavelength (approximately. 3.8 mm) in the 1 MHz chamber and 3.5 times the wavelength (approximately 2.5 mm) in the 2 MHz chamber. Chambers were modelled and filled with oil (Fig. S8, supporting information). All material properties in the simulation were matched to the real properties of the materials used in the chambers.

The simulation geometry was divided into a finite number of hexahedral elements with a maximum size of 1/8th of the wavelength by performing meshing. First, the standing acoustic field in frequency domain was solved by applying 25 V and 10 V peak-to-peak driving voltage to the active piezo element in the 1 MHz and 2 MHz chambers, respectively. Voltage amplitudes were selected to match the values used in the corresponding experiments. Second, particle movement was simulated in the time domain by calculating the effects of forces (acoustophoretic, drag, gravity) on 5,000 or 10,000 spherical particles of 100 µm diameter made of carbon or monostearin (selected as representative molecules for monoglycerides) randomly distributed in oil and subjected to the previously simulated standing acoustic field. In all experiments, the default solvers suggested by COMSOL were used.

### Schlieren imaging

The ultrasonic standing field in the experimental chambers filled with oil was imaged using a Schlieren setup. The imaging was performed using the setup described in Lampsijärvi et al.^47^ after some modification. Briefly, the Schlieren imaging setup was composed of a straight dual-lens Schlieren system (f1 = f2 = 200 mm, ø = 24.5 mm), an LED source (523 nm, LZ1-00G102, LED Engin, Inc., Wilmington, MA, USA), a digital camera (UI-3480CP, IDS Imaging, Obersulm, Germany) and a microtome blade mounted on a three-axis translation stage used as the spatial filter. The experimental chamber filled with oil was placed between the lenses. Pictures were taken before (background) and during sonication. The ultrasonic standing field generated between the transducers was obtained as described in the experimental chamber paragraph. The background was removed from all images using ImageJ software^48^, version 1.51a.

### Brightfield and polarized light real time microscopy

Microstructural changes during ultrasonic standing wave treatment were recorded using a digital microscope (Shenzhen Andonstar Tech Co., Ltd, Guangdong, China). Images and videos were recorded using Windows 10 built-in webcam application software. Polarized light images and videos were obtained using two LPVISE100-A polarized lenses (Thorlabs, Newton, NJ, USA) oriented at 90° with respect to each other and mounted on custom-made 3D-printed polylactic acid supports. Selected images were analyzed using ImageJ software^48^, version 1.51a. Images were converted to 8-bit grey, and then a line plot profile was recorded by drawing a straight line on the image after spatial calibration. Band thickness was calculated by drawing several straight lines on different dense crystalline bands.

### Synchrotron light X-ray diffraction

X-ray diffraction patterns were recorded at the X-ray diffraction beamline XRD1 at the Elettra Synchrotron Radiation Facility in Trieste (Italy). The X-ray beam emitted by the wiggler source on the Elettra 2 GeV electron storage ring was monochromatized by a Si(111) double crystal monochromator, focused on the sample and collimated by a double set of slits to a spot size of 50 × 50 µm^2^. The experimental chamber was mounted between two copper palates connected to a programmable water bath. Circulating water was used to control the cooling rate of the chambers during treatment. Analyses were performed during cooling from 80 °C to 20 °C at 1 °C min^−1^ and after the samples reached 20 °C. Samples were scanned vertically and horizontally using 50 µm steps. Data were collected at a photon energy of 8.856 keV (λ = 1.4 Å) using a 2 M Pilatus silicon pixel X-ray detector (DECTRIS Ltd., Baden, Switzerland). Bidimensional patterns collected with Pilatus were calibrated using lanthanum hexaboride powder (LaB_6_, standard reference material 660a of NIST) and integrated using the software FIT2D^49^, version 12.77. The indexing of the XRD patterns obtained by the crystalline phase was performed using the program WinPlotr^50^, version July 2017.

### Colorant migration test

We performed a colorant migration test on USSW, HIU, mechanically sheared and statically crystallized oleogel using the method described by Hwang, Fhaner, Winkler-Moser and Liu^45^. In this method, dyed oil is added on top of 5% monoglyceride oleogels cooled at 10 °C min^−1^, and the distance travelled by the colorant inside the oleogel is measured over time. All colorant test samples were obtained using 1 mL of molten oleogel. The oil was dyed with Oil Red O (Sigma-Aldrich).

To start, we upscaled the experimental chambers by increasing the volume from 150 µL (commonly used in other experiments) to more than 1 mL. The chambers comprised a cuvette with a path length of 10 mm in which the bottom part was replaced with Polyimide tape (thickness of 50 µm, Kapton tape, China). After the molten oleogel was poured into the cuvette, an aluminum reflector (2.5 mm thick) was placed above the sample at 1 cm distance from the bottom. Samples were sonicated at 1 MHz and 2 MHz with an input voltage of 25 V and 10 V (acoustic intensity < 1 W cm^−2^), respectively, by placing the cuvette on top of a transducer controlled by the custom-built firmware described above. To allow the transmission of ultrasound between the transducer and the cuvette, Aquasonic 100 ultrasound transmission gel (Parker Laboratories, Inc., Fairfield, NJ, USA) was used. The modified cuvette, aluminum reflector, gel, and transducer were preheated to 80 °C before starting the experiment. Sonication was done for 5 min after the sample turned opaque. In the case of 1 MHz sonication, a heat sink mounted with a fan was used to dissipate excess heat.

Unmodified cuvettes were used for the HIU treatment and for the sheared and statically crystallized samples. After the addition of the molten oleogel into the preheated cuvette at 80 °C, the sample was sonicated at 20 kHz using a Vibra-Cell HIU device operating at 130 W nominal electric power (Sonics & Materials Inc., Newtown, CT, USA) equipped with a 3.1 mm tip (intensity > 1 W cm^−2^). The intermittent sonication amplitude was 20% of the maximum amplitude of the HIU equipment with a total treatment time of 15 s and with intermittent 1 s ‘on’ and 1 s ‘off’ (parameters optimized to allow oleogels to crystallize without developing extra heat). The cuvette was immersed in a cold-water bath, and as soon as the oleogelator started to crystallize (visually determined) HIU was applied^22^. The tip was immersed into the forming oleogel, close to the surface.

Molten oleogel was added into the preheated cuvette and left to crystallize at room temperature with the same aluminum reflector as used in the US-SW-assisted crystallization experiment. The molten oleogel was added to the preheated cuvette. During cooling, the sample was sheared with a spatula at a frequency of two complete turns per second. The shearing was applied during the crystallization process and continued for 1 min after the sample turned completely opaque.

After samples were crystallized, they were left to rest for 30 min at − 20 °C. This operation was necessary to allow easy removal of the reflector (if present) and thus minimize damage to the sample surface. All samples were cooled at − 20 °C for 30 min to share the same thermal history. Following, sample were stored at room temperature for 24 h before analysis. Each sample was prepared in triplicate. Next, 1 mL of dyed oil was added on top of each sample, and colorant migration was carefully measured for 72 h at regular intervals with a caliper. Three experimental replicates were prepared for each treatment, and each sample at each time point was analyzed three times.

### Data analysis

All determinations were expressed as the mean ± standard deviation of at least two measurements from two experimental replicates (n ≥ 2 × 2) if not otherwise specified. Statistical analysis was performed using the software R, version 3.5.1^51^. A Bartlett’s test was used to check the homogeneity of variance, a one-way ANOVA was carried out and a Tukey’s test was used as a *post-hoc* test to determine significant differences among means (p < 0.05). Colorant migration data were fitted using the TableCurve 2D, version 5.01 (Jandel Scientific Software, San Rafael, CA, USA). Non-linear regression analysis was performed using (Eq. 8):

8 $$y=K\cdot {t}^{n},$$

where *y* is the distance in mm travelled by the colorant inside the oleogel, *K* is the migration rate constant, *t* is time, and *n* is the reaction order. The Levenberg–Marquardt algorithm was used to perform least squares function minimization, and the goodness of fit was evaluated based on statistical parameters of fitting (R^2^, p, standard error) and the residual analysis. Differences among estimated migration rate constants were determined using one-way ANOVA and a Tukey’s test, as described above.

## Supplementary information

Supplementary information.

Supplementary video legends.

Supplementary video 1.

Supplementary video 2.

Supplementary video 3.

## Abbreviations

USSW: Ultrasonic standing wave
HIU: High-intensity ultrasound
