# Supplementary material for: Controlling oleogel crystallization using ultrasonic standing waves
Source: Sci Rep. 2020 Sep 2;10:14448. doi: 10.1038/s41598-020-71177-6 (PMC7468300; doi:10.1038/s41598-020-71177-6)
Supplement: Supplementary file 1 — Supplementary information. [file 41598_2020_71177_MOESM1_ESM.pdf]

SUPPORTING INFORMATION

Controlling oleogel crystallization using ultrasonic standing waves

Fabio Valoppi[1,2,3*], Ari Salmi[3], Miika Ratilainen[3], Luisa Barba[4], Tuomas Puranen[3], Oskari Tommiska[3], Petteri Helander[3], Jesse Heikkilä[3], Edward Haeggström[3]

Affiliations:

[1] Department of Food and Nutrition, P.O. Box 66 (Agnes Sjöbergin katu 2), FI-00014 University of Helsinki, Finland

[2] Helsinki Institute of Sustainability Science, Faculty of Agriculture and Forestry, FI-00014 University of Helsinki, Finland

[3] Electronics Research Laboratory, Department of Physics, P.O. Box 64 (Gustaf Hällströmin katu 2), FI-00014 University of Helsinki, Finland

[4] Istituto di Cristallografia, Consiglio Nazionale delle Ricerche, 34100 Trieste, Italy

* corresponding author

Phone: +358294158223, email: fabio.valoppi@helsinki.fi

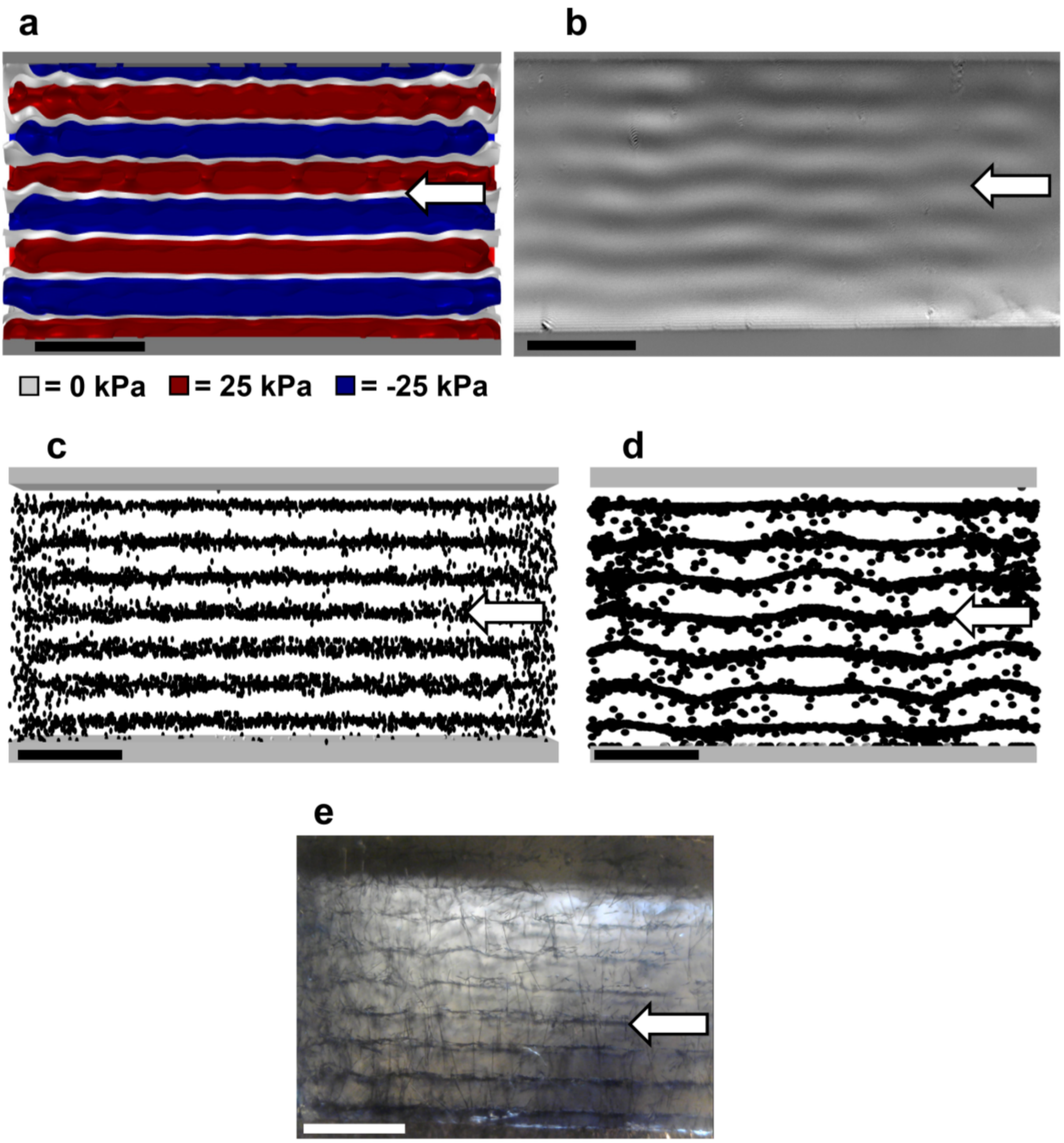

Figure S1. (**a**) Finite element simulation of the ultrasonic standing field in the 2 MHz experimental chamber (top and bottom gray parts are the piezoceramic elements). (**b**) Schlieren imaging of the USSW field in the 2 MHz experimental chamber, with oil used as the medium. (**c**) Frontal view (XZ plane) of the particle movement simulation for 10,000 spherical particles of 100 μm diameter made of monostearin after 10 s in the 2 MHz USSW field. Boundary effects are visible at the lateral sides of the simulated experimental chamber. (**d**) Corresponding lateral view (YZ plane) of (**c**). (**e**) Photograph of 30 μm–175 μm long carbon rods dispersed in rapeseed oil and subjected to a 2 MHz USSW field. Scale bars represent 1 mm in the vertical direction. The arrow indicates the same pressure node across all images.

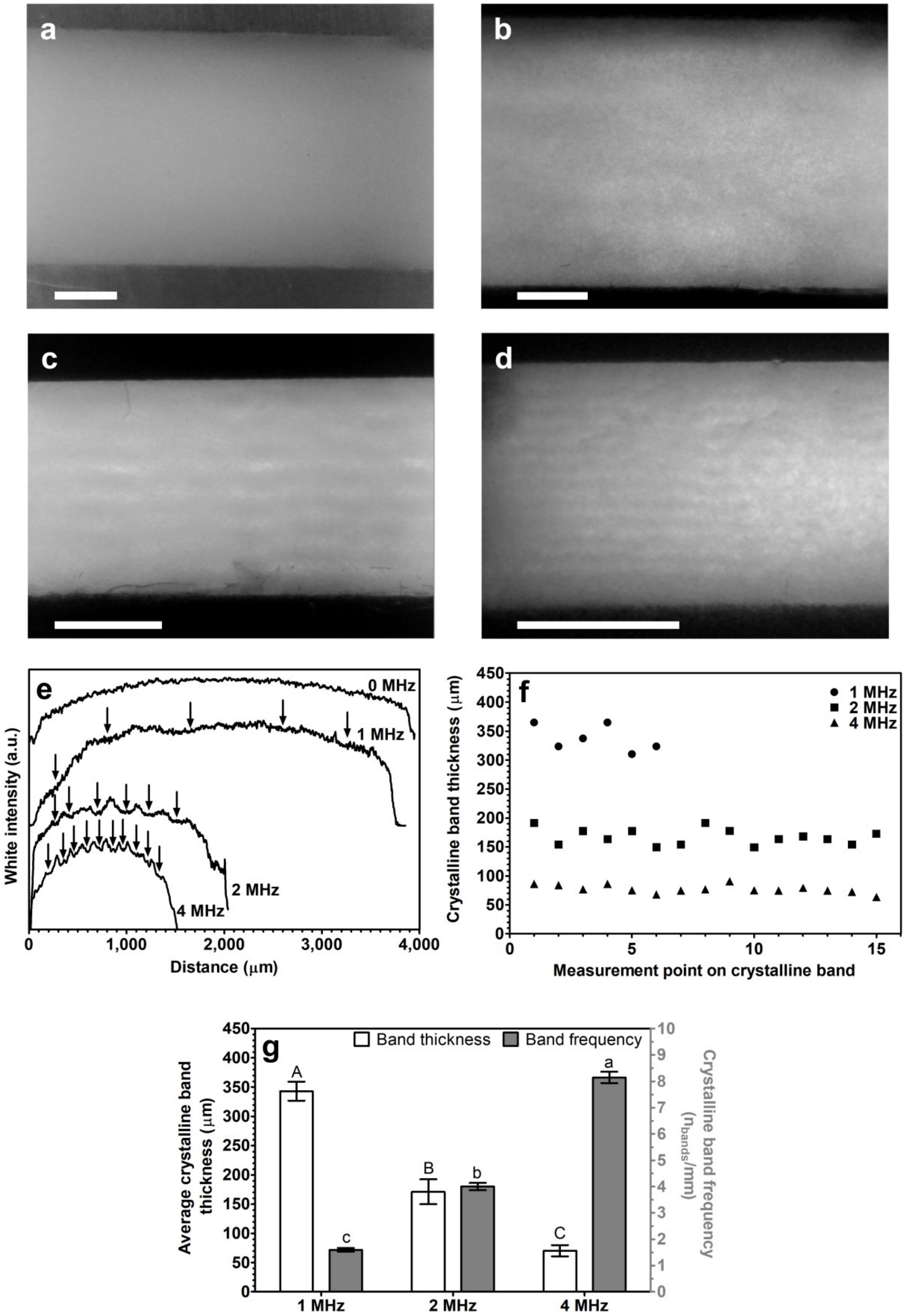

Figure S2. (**a**) Photograph: oleogel statically crystallized, control sample (0 MHz). (**b**) Photograph: oleogel crystallized in a 1 MHz US-SW field. (**c**) Photograph: oleogel crystallized in a 2 MHz US-SW field. (**d**) Photograph: oleogel crystallized in a 4 MHz US-SW field. (**e**) White intensity recorded in the Z direction (vertically) from a photograph as a function of the distance from the top transducer for the

control sample (0 MHz) and the sonicated samples (1 MHz, 2 MHz, and 4 MHz). Curves were shifted vertically for clarity. The arrows indicate the presence of a crystalline band. (**f**) Thickness of a crystalline band measured on 15 points in the X direction (horizontally) from a photograph of the sonicated samples (1 MHz, 2 MHz, and 4 MHz). (**g**) Average crystalline band thickness and normalized number of crystalline bands per mm for the sonicated samples (1 MHz, 2 MHz, and 4 MHz) analyzed from photographs. Data is expressed as mean ± standard deviation based on n = 2 experimental replicates × 8–12 repeated measurements for the band thickness and n = 2 experimental replicates for the crystalline band frequency. Values with different uppercase or lowercase letters are statistically different ($p < 0.05$). In (**a**), (**b**), (**c**), and (**d**), oleogels contain 5% monoglyceride in rapeseed oil and are crystallized at 10 °C/min. The top and bottom dark areas are the piezoceramic transducers, and the scale bars are 1 mm (both vertical and horizonal directions).

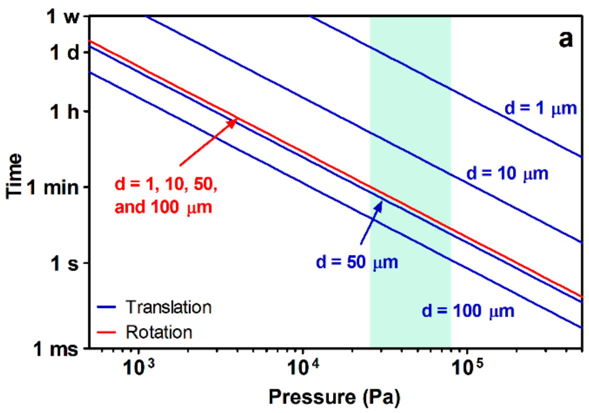

Figure S3. Analysis of simulation model. Theoretical estimated characteristic time for the rotation (orientation, t_{rot}) and translation (t_{tra}) of disk-shaped monostearin crystals with a diameter of 1 μm, 10 μm, 50 μm, and 100 μm and an aspect ratio (d/h) equal to or greater than 10 in (**a**) a 2 MHz (2012 kHz) US-SW field and (**b**) a 4 MHz (4120 kHz) US-SW field. The highlighted area corresponds to the estimated pressure amplitude based on the time needed during experiments to form bands, taking into consideration platelets that are 50 μm–100 μm in size.

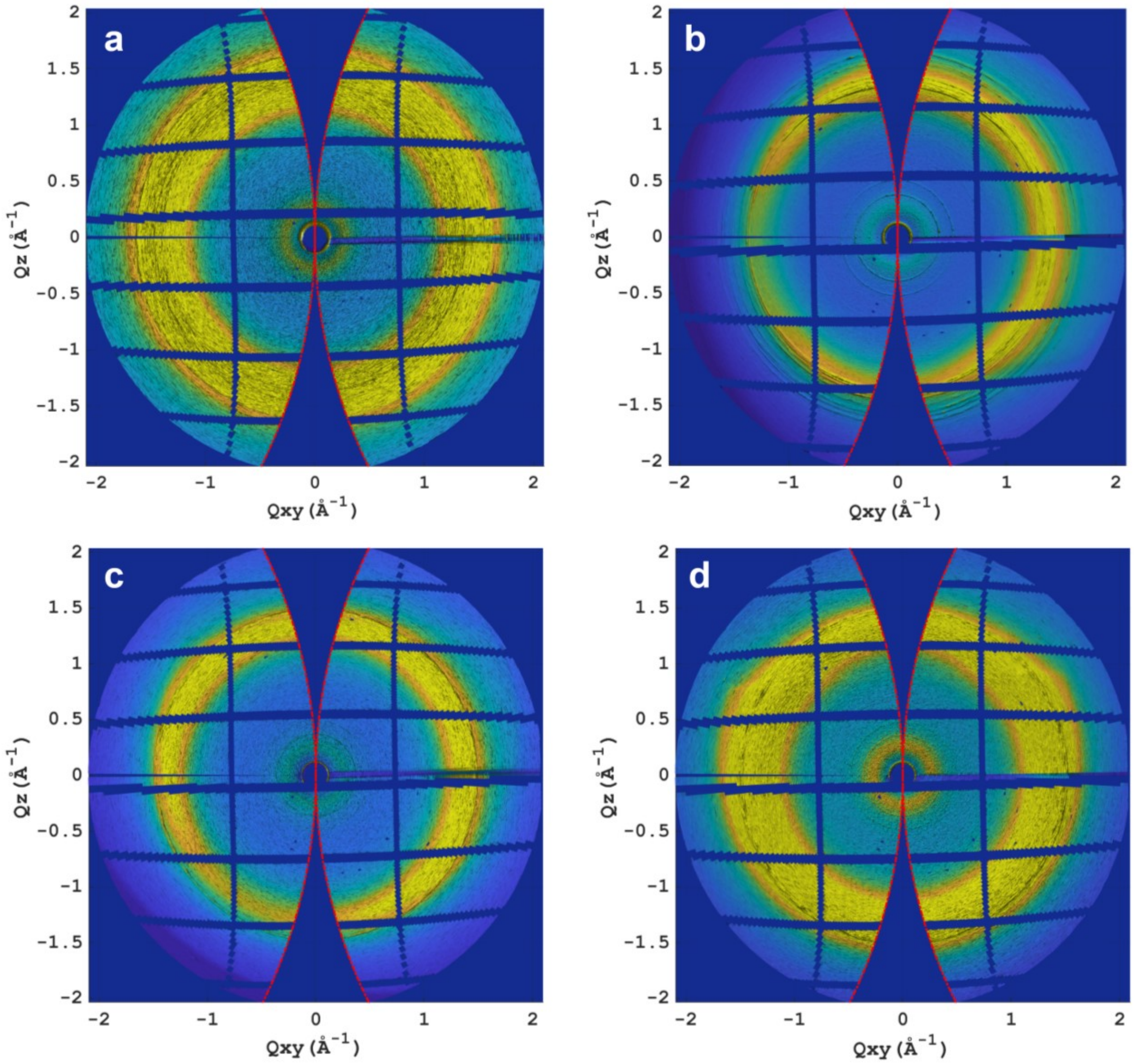

Figure S4. Experimental data. Diffraction patterns expressed as intensity in the Qxy-Qz plane of (**a**) oleogel statically crystallized, control sample (0 MHz); (**b**) oleogel crystallized in a 1 MHz US-SW field; (**c**) oleogel crystallized in a 2 MHz US-SW field; and (**d**) oleogel crystallized in a 4 MHz US-SW field. All samples contain 5% monoglyceride in rapeseed oil and are crystallized at 1 °C/min. Q represents the reciprocal lattice vector; its modulus $|Q| = 2\pi/d = (4\pi/\lambda)\cdot\sin(\theta)$ is the reciprocal lattice spacing, where d is the lattice spacing, λ is the X-ray wavelength, and 2θ is the Bragg scattering angle.

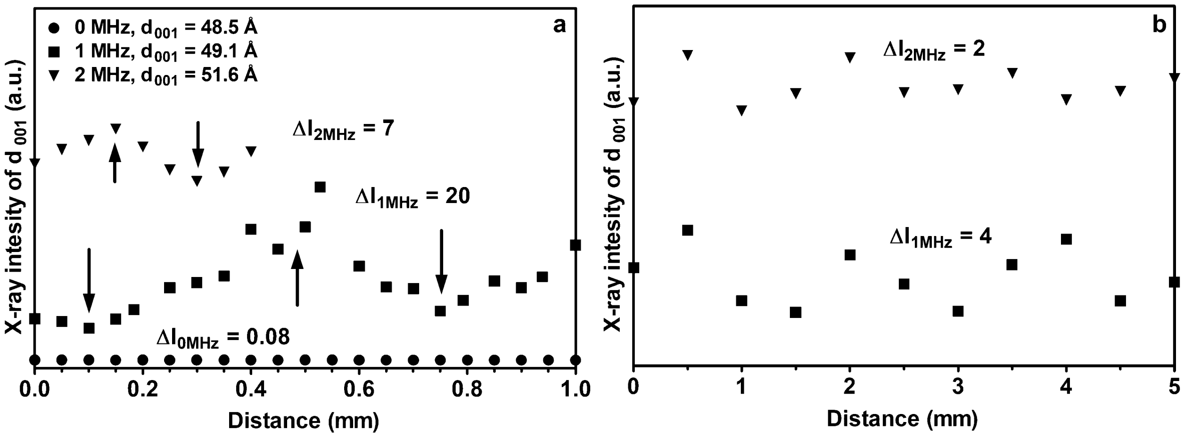

Figure S5. Experimental data. (**a**) X-ray intensity recorded on the Debye–Scherrer ring in the small-angle region corresponding to the 001 reflection of the monoglyceride lamellar structure (d_{001}) as a function of distance during vertical scans for oleogel statically crystallized, control sample (0 MHz), and oleogels crystallized in a 1 MHz US-SW field and a 2 MHz US-SW field. Arrows indicate nodal (upward) and anti-nodal (downward) planes. (**b**) X-ray intensity recorded on the Debye–Scherrer ring in the small-angle region corresponding to the 001 reflection of the monoglyceride lamellar structure (d_{001}) as a function of distance during horizontal scans on the crystalline bands showing the maximum intensity for (a) oleogels crystallized in a 1 MHz US-SW field and a 2 MHz US-SW field. All samples contain 5% monoglyceride in rapeseed oil and are crystallized at 1 °C/min.

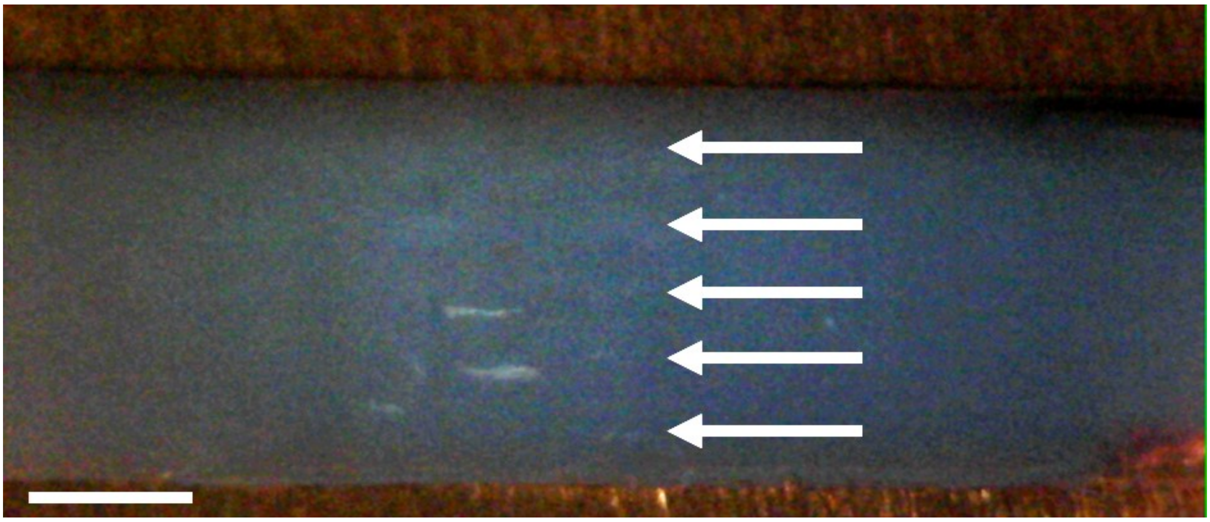

Figure S6. Polarized light micrograph showing the formation of bands in oleogels containing 5% candelilla wax subjected to a 2 MHz US-SW field crystallized at 10 °C/min. The top and bottom dark areas are the piezoceramic transducers, and the scale bar is 1 mm.

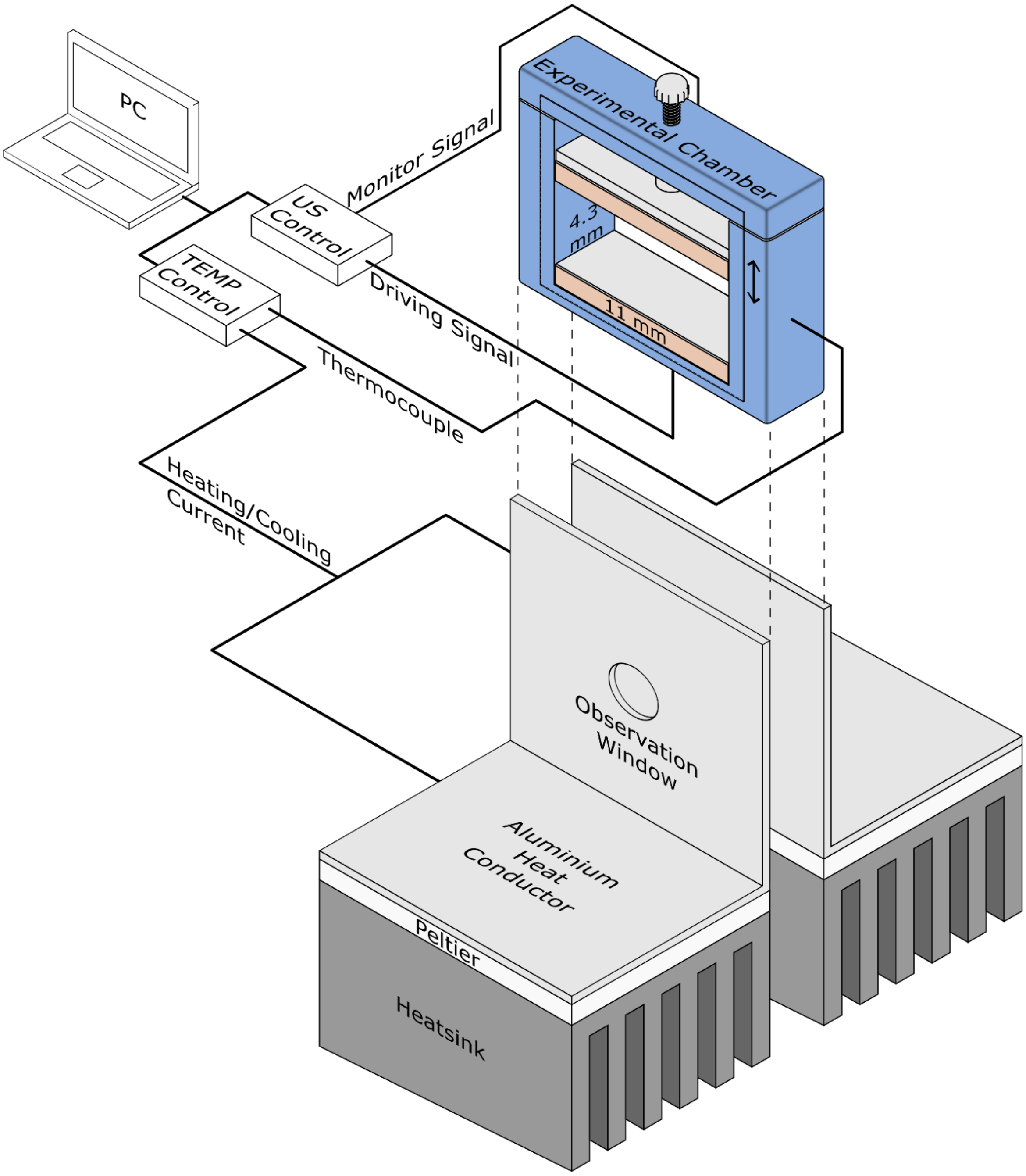

Figure S7. Schematic of the experimental setup featuring the experimental chamber, the temperature control device, and the hardware to control the ultrasound and temperature.

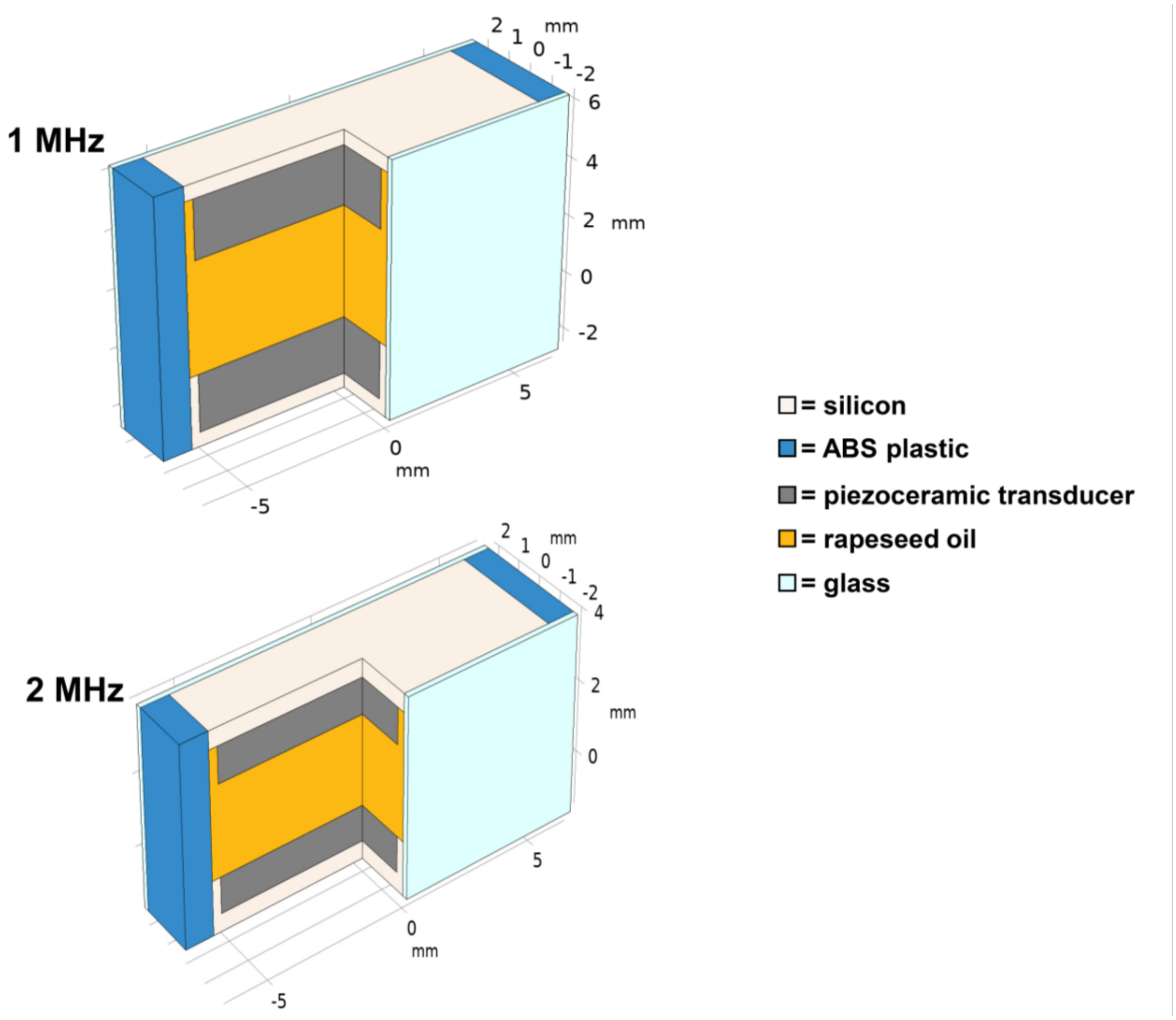

Figure S8. Schematics of the geometries used in FEM simulations for the 1 MHz and 2 MHz chambers. Part of the geometry is cut to show the internal parts. Materials are indicated by different colors.

Mathematical derivation of the dynamic behavior of crystalline platelets in oil subjected to a USSW field

The mathematic modelling of small particles immersed in an ultrasonic field is relatively straightforward. The particle behavior is defined by the acoustophoretic force and the drag force on the particle. We assume that the particles are much smaller than the wavelength of the ultrasonic standing wave (US-SW). By considering one crystal at a time and by ignoring boundary effects, we reduce the problem to one dimension, z. By limiting ourselves to particles that are disk-shaped, the orientation of the crystal can be described with a single angle θ, which describes the rotational offset from the equilibrium orientation. The model considers neither the acoustic streaming nor the coupled flow of the fluid-crystal two-phase system. These phenomena require FEM modeling and are outside the scope of this analysis.

The equations of motion of the system are:

$$m\frac{\partial^2 z}{\partial t^2} = F_{\text{drag}} + F_{\text{a}} \text{ and}$$

$$I\frac{\partial^2 \theta}{\partial t^2} = \tau_{\text{drag}} + \tau_{\text{a}} \,,$$

with the first being for transport and the second for orientation. Due to their small size, the crystals exhibit negligible inertia compared to the viscous forces. Consequently, the equations reduce to:

$$B\frac{\partial z}{\partial t} = K_a \sin 2kz \text{ and } \beta\frac{\partial \theta}{\partial t} = \kappa_{\text{a}} \sin 2\theta.$$

Here, B and β are the drag coefficient for transport and rotation, respectively. We write the orientation and position dependency on the acoustic torque and force. All coefficients have many dependent variables—crystal size, crystal shape, and standing wave pressure. The two equations are coupled because the acoustic force depends on the orientation and the acoustic torque and on the position in the wave. However, because these dependencies are weak and because the two phenomena occur at different time scales, we treat the two equations as separate. This way, we arrive at an effective approximation.

The detailed form of the acoustic force and torque on immersed ellipsoids is given by Silva and Drinkwater[1] and Fan, Mei, Yang and Chen[2].

$$F_{\text{a}} = \frac{5}{4}V_p\rho\left(\frac{p}{\rho c}\right)^2 k\sin(kz)\cos(kz)\left(1-\frac{1}{5}\cos 2\theta\right)$$

$$\tau_{\text{a}} = \frac{1}{4}V_p\rho\left(\frac{p}{\rho c}\right)^2 \sin^2 kz\left[\left(\frac{1+k_3}{\rho_{\text{p}}+k_3\rho} - \frac{1+k_1}{\rho_{\text{p}}+k_1\rho}\right)(\rho-\rho_{\text{p}})\right]\sin 2\theta$$

Here, V_p is the crystal volume, ρ is the density of the medium, p is the acoustic pressure where the particle is, ρ_p is the density of the crystal, k is the acoustic wavenumber, and c is the speed of sound (phase velocity) in the medium. Factors k_1 and k_3 are values of added mass that describe the increase in the effective mass due to the inertia of the surrounding medium. Due to the assumption of a large aspect ratio, $k_1 \gg k_3$.

The drag coefficients are trickier. Ortega and de la Torre[3] provide simulation results for diffusion coefficients of disk-shaped objects. The diffusion coefficients, defined as $D_t = k_{\text{B}}T/B$ and $D_r = k_{\text{B}}T/\beta$, depend on the disk aspect ratio p. The authors define three rotational times:

$$\tau_a = \frac{1}{6D_r^{\perp}};\ \tau_b = \frac{1}{(5D_r^{\perp}+D_r^{\parallel})};\ \tau_c = \frac{1}{(2D_r^{\perp}+4D_r^{\parallel})},$$

from where the rotational diffusion coefficient $D^{||}$ can be solved. The orientation times are:

$\tau_a/\tau_0 = 1.18 + 0.1744(\ln a + 0.2877)^2 - 0.2417(\ln a + 0.2877)^3 - 3.882\times10^{-2}(\ln a + 0.2877)^4$

$\tau_b/\tau_0 = 1.183 + 0.2902(\ln a) + 0.4406(\ln a)^2 - 5.850\times10^{-2}(\ln a)^3 - 9.544\times10^{-3}(\ln a)^4$

$\tau_c/\tau_0 = 0.9833 + 6.532\times10^{-2}/a + 5.168\times10^{-2}/a^2 - 3.234\times10^{-3}/a^3$

$$\tau_0 = \frac{\pi L^3 \eta_0}{4p^2 kT}.$$

The translational diffusion coefficient is defined as:

$f/f_0 = 1.009 + 1.395\times10^{-2}(\ln p) + 7.880\times10^{-2}(\ln p)^2 + 6.040\times10^{-3}(\ln p)^3$

$f_0 = 6\pi\eta_0 L(3/16p^2)^{1/3}$.

One sees that translational drag is proportional to the first power of the crystal diameter, whereas rotational drag is proportional to the third power. One also sees the different dependence on acoustic pressure for translation and rotation.

Once all coefficients are known, the differential equation can be solved. We focus on the rotational equation, as both equations feature a similar dependency. In a case where β and κ stay constant over time, the equation has an analytical solution, $\theta(t) = \cot^{-1}(Ce^{-2\kappa t/\beta})$. This assumption holds when the rotation happens faster than the crystal growth, so the model only works for the shortest rotation times. If we assume a uniform distribution for the initial angle, the median time for a crystal to orientate to within 5 degrees from equilibrium is:

$$t_{\mathrm{rot}} = \frac{\beta}{2\kappa} \ln\left(\frac{\tan 45}{\tan 5}\right) \approx 1.2 \cdot \beta/\kappa.$$

Because the translational equation has the same form, it has similar solution.

The model could be further developed by considering both equations simultaneously and by considering the crystal growth.

Symbols

p	acoustic pressure
ρ	density of oil 910 g/l
ρ_{p}	density of crystal 1030 g/l
c	speed of sound in oil
μ	viscosity
a	crystal aspect ratio
V	crystal volume
z	coordinate along the standing wave
θ	crystal orientation, measured as deflection from equilibrium
k	wave number
k_1	value of added mass, parallel to disk axis
k_3	value of added mass, perpendicular to disk axis
