# Supplementary material for: Controlling oleogel crystallization using ultrasonic standing waves
Source: Sci Rep. 2020 Sep 2;10:14448. doi: 10.1038/s41598-020-71177-6 (PMC7468300; doi:10.1038/s41598-020-71177-6)
Supplement: Supplementary file 2 — Supplementary video legends. [file 41598_2020_71177_MOESM2_ESM.docx]

**Video Legends**

**Video 1.** Frontal view (XZ plane) of particle movement simulation for 10,000 spherical particles of 100 µm diameter made of monostearin subjected to a 1 MHz USSW field. The distance between piezo elements is approximately 3.8 mm. The accumulation of the particles in the nodal planes is visible. Boundary effects are visible at the lateral sides of the simulated experimental chamber.

**Video 2.** Frontal view (XZ plane) of particle movement simulation for 10,000 spherical particles of 100 µm diameter made of monostearin subjected to a 2 MHz USSW field. The distance between piezo elements is approximately 2.5 mm. The accumulation of the particles in the nodal planes is visible. Boundary effects are visible at the lateral sides of the simulated experimental chamber.

**Video 3.** Oleogel formation in a 1 MHz USSW field during cooling at 10 °C/min recorded using real-time polarized light microscopy. The bright areas correspond to the forming of monoglyceride crystals, while the black areas correspond to rapeseed oil. Tilting and accumulation of the crystals in the nodal planes are visible.
